# Recent Advances in Brain Physiology and Cognitive Processing****

**DOI:** 10.4103/0973-1229.77434

**Published:** 2011

**Authors:** Pereira Alfredo, Maria Alice Ornellas Pereira, Fábio Augusto Furlan

**Affiliations:** * *Adjunct Professor on Philosophy of Science, Institute of Biosciences, State University of São Paulo (UNESP), Campus Rubião Jr., 18618-000, Botucatu-SP, Brasil*; ** *Adjunct Professor on Psychiatric Nursing, Faculty of Medicine, State University of São Paulo (UNESP), Botucatu-SP*; *** *Professor of Physiology, Faculty of Medicine and Nursing, University of Marília, Marília-SP*; **** *Revised and peer reviewed version of a paper for an International Seminar on Mind, Brain, and Consciousness, Thane College Campus, Thane, India, January 13-15, 2010.*

**Keywords:** *Astrocyte*, *Calcium Waves*, *Glutamate*, *Tripartite Synapse*

## Abstract

The discovery of participation of astrocytes as active elements in glutamatergic tripartite synapses (composed by functional units of two neurons and one astrocyte) has led to the construction of models of cognitive functioning in the human brain, focusing on associative learning, sensory integration, conscious processing and memory formation/retrieval. We have modelled human cognitive functions by means of an ensemble of functional units (tripartite synapses) connected by gap junctions that link distributed astrocytes, allowing the formation of intra- and intercellular calcium waves that putatively mediate large-scale cognitive information processing. The model contains a diagram of molecular mechanisms present in tripartite synapses and contributes to explain the physiological bases of cognitive functions. It can be potentially expanded to explain emotional functions and psychiatric phenomena.

## Introduction

Although astrocytes compose one-half of brain tissue volume, until recently only passive functions were attributed to these star-shaped cells, such as giving structural, metabolic and functional support for neurons. However, a growing number of ‘in vitro’ and ’in vivo’ results support the conception that astrocytes are also excitable cells and play important roles in information processing in the brain (Perea and Araque, 2005; Haydon and Carmignoto, 2006; Schummers *et al*., 2008; Wang and Bordey, 2008).

Recent research focusing on the participation of astrocytes in glutamatergic synapses has revealed a connection between the following four human cognitive functions: learning, perception, conscious processing and memory formation/retrieval.

## The Glutamatergic Tripartite Synapse

**Figure 1:**
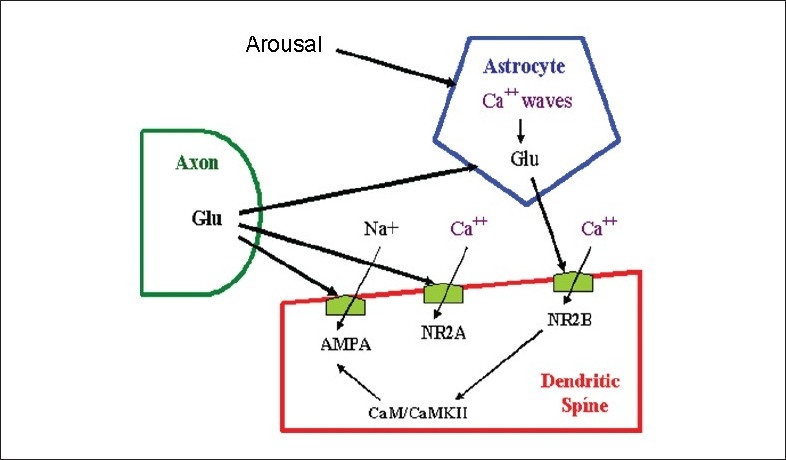
The glutamatergic tripartite synapse. Glutamate (Glu) released by the presynaptic neuron binds with both astroglial (MGluR) and postsynaptic neuronal (AMPA and NR2A) receptors. MGluR activate the inositol triphosphate (IP3) pathway, inducing the release of calcium ions from internal stores (mitochondria and endoplasmatic reticulum) to prompt Glu release. Astroglial Glu binds mostly with neuronal NMDA receptors containing the NR2B subunit (NR2B), causing calcium ion entry (slow inward currents) and then sustaining excitatory activity of the postsynaptic neuron (adapted from Pereira and Furlan, 2009).

Associative learning and memory formation are classically illustrated at the synaptic level by means of a model composed of two (the pre- and postsynaptic) connected neurons, and their respective inter- and intracellular signalling pathways. The discovery of the participation of astrocytes as active elements in synaptic transmission/computation has led to the construction of broader models, composed by functional units of two neurons and one astrocyte, the tripartite synapses [Figure 1].

Astrocyte terminations wrap the synaptic cleft. In some brain regions, each astrocyte can contact up to 140,000 synapses (Agulhon *et al*., 2008), respond to presynaptic input by means of calcium waves and release gliotransmitters that modulate neural activity.

## The Astroglial Network

**Figure 2:**
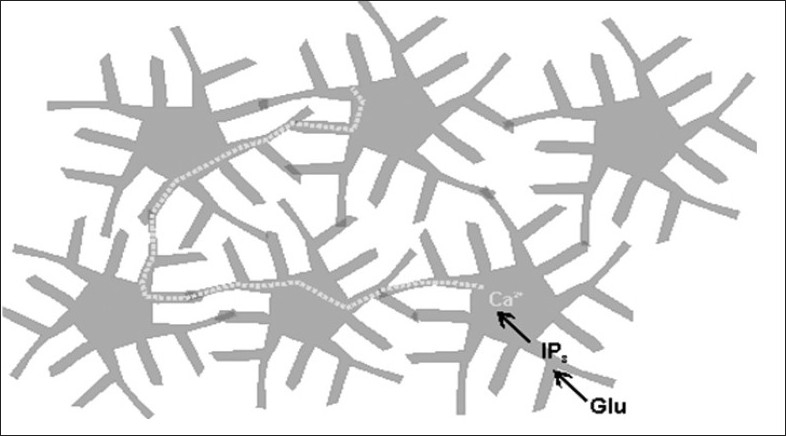
Calcium Waves in the Astrocytic Syncytium. The activation of calcium waves in one astrocyte, by means of the binding of neuronal Glutamate, with a metabotropic receptor activating the inositol triphosphate pathway, releases calcium ions from the endoplasmatic reticulum, causing a “saltatory” movement (Figure adapted from http://synapses.mcg.edu).

Neighbouring astrocytes are coupled by gap junctions forming a functional syncytium [Figure 2].

Human cognitive functions, including conscious perception, can be modelled by an ensemble of tripartite synapses connected by the astrocytic syncytium (Pereira Jr and Furlan, 2009). This kind of model can be useful to explain the cognitive roles of both short- and long-term potentiation and depression, as well as calcium waves in astrocytes.

## Sketch of a Model of Cognitive Functions of the Astroglial Network

**Figure 3:**
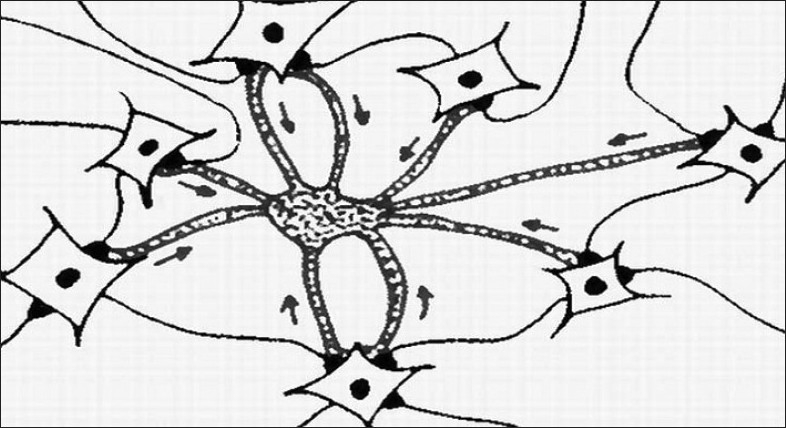
Induction of a calcium wave in one astrocyte, by means of coordinated excitatory input from a surrounding neuronal population. Arrows indicate concentric excitation promoted by synchronised neurons impacting the target astrocyte (Adapted from Pereira Jr and Furlan, 2009).

The model contributes to explain the physiological bases of cognitive functions according to the following stages:

- Glutamatergic heterosynaptic converging input to a neocortical or hippocampal neuron activates AMPA receptors and the resulting depolarization opens NMDA receptors of the NR2A subtype, promoting calcium ion entry that cause membrane potentiation related to associative learning (mostly by means of a signalling cascade and gene expression that leads to an increase in AMPA-dependent response);
- At the same time, the glutamatergic input activates metabotropic receptors in the membrane of one single astrocyte that wraps almost all such active synapses;
- When such local glutamatergic converging input are synchronised, the resulting (additive) stimulation over the membrane of the astrocyte crosses a given threshold and elicits coherent, amplitude- and/or frequency-modulated calcium waves with the potential of integrating local information [Figure 3];
- When global brain synchronisation occurs, calcium waves integrate sensory, cognitive and affective/emotional patterns from distinct neuronal populations;
- Glutamate released from astrocytes to postsynaptic neurons in tripartite synapses binds to extrasynaptic NMDA receptors of the NR2B subtype, which drives slow inward calcium currents, causing a delayed depolarization and an increase of CaMKII phosphorylation and AMPA excitability, a process we called “meta-potentiation” (Pereira Jr and Furlan, 2007) and reinforces long-term potentiation, or alternatively triggers a process of long-term depression.

## Neuroastroglial Interactions

The dynamical process that boosts neuroastroglial communication is, according to our model, the synchronisation of neuronal graded and action potentials. Synchronisation of large populations of neurons, in several medium to high frequencies (from theta to gamma), increases glutamate release from neurons to astrocytes, binding with metabotropic receptors that elicit astrocytic calcium waves by means of an activation of the inositol triphosphate pathway.

According to a model developed by De Pittà *et al*. (2008), the dynamics of these waves may encode information about external stimuli in amplitude and/or frequency modulation. Frequency-modulated waves shift to amplitude-modulated when the excitation reaches a threshold and the state of inositol triphosphate activity inducing calcium release reaches a fixed point. The amplitude-modulated locally generated waves cross the astrocytic syncytium and promote an integration of the information embodied in the populations of neurons connected to the astrocytes. The dynamics of calcium waves is “saltatory” according to Roth *et al*. (1995), producing fast integration of patterns along a population of cells.

We have made (Pereira Jr., 2007) an approximation of this model with the prospects of a large-scale Ion-Trap Quantum Computer (ITQC) proposed by Kielpinski *et al*. (2002), to be combined with the proposal of a “magnetic dialogue” between neurons and astrocytes (Banaclocha, 2007). This approximation takes into account the similarity of the ITQC with the dynamics of calcium ions trapped inside astrocytic membranes previously hyperpolarised by the movement of potassium ions (Wang and Bordey, 2008) and affected by neuronal action potentials (Postnov *et al*., 2007).

## The Astrocentric Hypothesis

**Figure 4:**
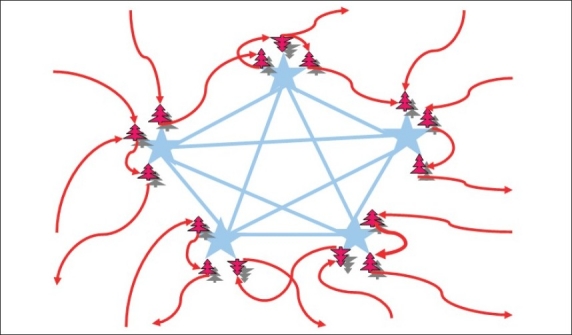
A Network of Astrocytes Participating in Tripartite Synapses. Blue stars represent astrocytes and red trees represent neurons. Each astrocyte participates in a tripartite synapse, being activated by the presynaptic neuron and contributing to sustain or reduce the activity of the postsynaptic neuron. Analogously to the process of generating a hologram, when neuronal potentials oscillations synchronise, calcium waves in astrocytes are more likely to form coherent patterns of activity. [Adapted from Pereira and Furlan, 2010]

According to the “Astrocentric Hypothesis” advanced by Robertson (2002; for detailed comments on the hypothesis, please check his site: http://artificialingenuity.com), conscious perception of a stimulus occurs when astrocytic calcium waves integrate neuronal distributed information patterns. In a theoretical perspective, the astrocytic syncytium can be viewed as a “Global Workspace” (according to the model presented by Baars, 1997) that integrates patterns from local neuronal assemblies to a brain-wide network [Figure 4], where it is broadcasted and made accessible to other local assemblies.

Once a conscious coherent process is formed in the brain’s astrocytic network, the resulting integrated information can feedback on brain activity, inducing effects on perceptual, cognitive, endocrine and motor systems. Such a feedback is carried from astrocytes to postsynaptic neurons by the slow inward calcium currents (driven by activation of extrasynaptic NR2B subtype NNDA receptors), causing depolarizations that impact on brain activity and behaviour.

Abnormalities in neuro-astroglial cross-talk may result in abnormal brain activity. In fact, astrocytes are involved in epileptic seizures (Silchenko and Tass, 2008; Reyes and Parpura, 2008), schizophrenia (Halassa *et al*., 2007) and depression (McNally *et al*., 2008), among other neurological and psychiatric phenomena (De Keyser *et al*, 2008), most of them accompanied by changes in conscious activity. The onset and propagation of pathological states can also be related to purinergic transmission (Verkhrasky *et al*., 2009).

**Figure 5:**
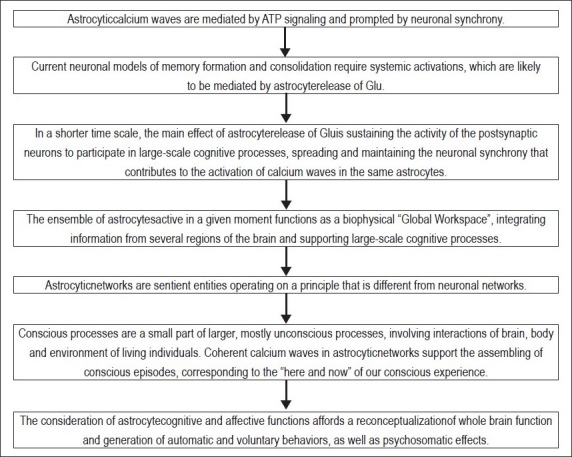
Flowchart of paper

We further suggest that conscious processing mediated by astrocytic calcium waves has a role in the determination of which patterns are more likely to form new memories that can be retrieved later. When a cognitive pattern is reinforced by astrocytic glutamatergic output to NMDA receptors, the chance to form long-term memories and be retrievable in the future increases. Correspondingly, the chance decreases if the pattern is “vetoed” by means of membrane depression. Postsynaptic neuronal membrane potentiation and depression are influenced by conscious processing, having an effect on memory and behaviour. Steps of the whole process are described in flowchart of paper [Figure 5]. For details of this model, see Pereira Jr. and Furlan (2010).

## Concluding Remarks

We strongly suggest that the astroglial network, together with other glial cells, blood and cerebrospinal fluid, constitutes a brain-wide signalling circuit supplementary to neuronal networks.

Physiologically, astrocytes can continuously communicate with each other by means of calcium waves and transport of small molecules. We hypothesise that this parallel network integrates spatially distributed neuronal information by means of interference of calcium waves generated at many cerebral locations from neuronal excitatory (glutamatergic) transmission.

Psychologically, we suggest that the astroglial network, besides integrating neuronal information, also generates a *feeling* about the content of this information. This astroglial function makes us cognitively different from information-processing machines as digital computers. Our cognitive processes are dynamically modulated by our feelings about what happens in our body and environment. This fact is of capital importance for theories in Psychiatry and for the practice of Mental Health professionals.

We understand conscious processing as the result of neuro-astroglial interactions. There is a division of work in the brain, such that neurons process information and astrocytes generate an affective response about the information. Therefore, in this model, consciousness is understood as a feeling about the content of information processed by the brain.

### Take home message

In light of recent advances in brain sciences, cognitive and affective functions cannot be attributed only to the activity of neurons.

The astroglial network has physiological and psychological functions different from neuronal networks.

Conscious agents develop cognitive operations different from machines, because the existence of consciousness implies an affective modulation of cognitive processes and their impact on (overt) behaviour and somatic processes.

## Questions That This Paper Raises

1. Does the astrocytic network operate as a Master Hub for brain-wide information integration?
2. Is the astroglial network the final target of general anaesthetics like Halothane and Ketamine?
3. How are mental diseases and illnesses related to astroglial dysfunctions?
4. What is the weight of astroglial activity on measurements of brain activity made with scalp electroencephalogram (EEG) and blood oxygenation-based functional magnetic resonance imaging (BOLD fMRI)?
5. Are calcium waves in astrocytes impacted by weak transcranial direct current stimulation (weak tDCS)? Do these waves participate in the generation of event-related potentials (ERPs)? How are they related to the activity of ‘default networks’?
6. Considering that current techniques of astroglial calcium waves imaging are based on fluorescence of calcium receptors in transgenic animals and require opening a hole in the skull, how could new noninvasive techniques be developed to allow ‘in vivo’ imaging in human subjects?
7. What are the prospects for psychiatric drugs that target glial functions? Would they bring the possibility of cure for most mental illnesses?

## About the Author

*Alfredo Pereira Jr. has a Master and a PhD in Philosophy of Science. During 1996 to 1998, he was a Post-Doctoral Fellow with Dr. Steve Chorover at the Dept. of Brain and Cognitive Sciences of the Massachusetts Institute of Technology. He has done research in Psychological Physiology, receiving since 2002 a grant from the Brazilian Research Council. Recently, he has specialised on cognitive function of astrocytes.*

## About the Author

*Maria Alice Ornellas Pereira has a Master and PhD in Psychiatric Nursing. During the year of 1995, she stayed in Trieste and Milan (Italy) for doctoral research on the Italian psychiatric reform, since then adopting Benedetto Saraceno’s approach to Psychosocial Rehabilitation. She worked in three Brazilian universities, including the School of Nursing of the University of São Paulo in Ribeirão Preto (USP) and the State University of São Paulo in Botucatu (UNESP).*

## About the Author

*Fábio Augusto Furlan is a biologist, PhD by São Paulo State University, Brazil, and a Professor of Physiology and Morphophysiology at University of Marília, Brazil. His research activity aims at the development of a model of cognitive functions based on the interaction between neurons and astrocytes in the brain.*
